# Metformin attenuates renal tubulointerstitial fibrosis via upgrading autophagy in the early stage of diabetic nephropathy

**DOI:** 10.1038/s41598-021-95827-5

**Published:** 2021-08-11

**Authors:** Fengzhen Wang, Haihan Sun, Bangjie Zuo, Kun Shi, Xin Zhang, Chi Zhang, Dong Sun

**Affiliations:** 1 School of Pharmacy, Xuzhou Medical University, Xuzhou, China; 2 Department of Pharmaceutics, Affiliated Hospital of Xuzhou Medical University, 99 West Huai-hai Road, Xuzhou, China; 3 Department of Internal Medicine and Diagnostics, Xuzhou Medical University, Xuzhou, China; 4 Department of Nephrology, Yancheng Third People’s Hospital, Yancheng, China; 5 Department of Orthopedics, Xuzhou Central Hospital, Xuzhou, China; 6 Department of Nephrology, Affiliated Hospital of Xuzhou Medical University, 99 West Huai-hai Road, Xuzhou, Jiangsu China; 7 Department of Nephrology, Affiliated Suqian Hospital of Xuzhou Medical University, Suqian, China

**Keywords:** Diseases, Medical research, Molecular medicine, Nephrology

## Abstract

This study aimed at comparing the effects of metformin on tubulointerstitial fibrosis (TIF) in different stages of diabetic nephropathy (DN) in vivo and evaluating the mechanism in high glucose (HG)-treated renal tubular epithelial cells (RTECs) in vitro. Sprague–Dawley (SD) rats were used to establish a model of DN, and the changes of biochemical indicators and body weight were measured. The degree of renal fibrosis was quantified using histological analysis, immunohistochemistry, and immunoblot. The underlying relationship between autophagy and DN, and the cellular regulatory mechanism of metformin on epithelial-to-mesenchymal transition (EMT) were investigated. Metformin markedly improved renal function and histological restoration of renal tissues, especially in the early stages of DN, with a significant increase in autophagy and a decrease in the expression of fibrotic biomarkers (fibronectin and collagen I) in renal tissue. Under hyperglycemic conditions, renal tubular epithelial cells inactivated p-AMPK and activated partial EMT. Metformin-induced AMPK significantly ameliorated renal autophagic function, inhibited the partial EMT of RTECs, and attenuated TIF, all of which effectively prevented or delayed the onset of DN. This evidence provides theoretical and experimental basis for the following research on the potential clinical application of metformin in the treatment of diabetic TIF.

## Introduction

Diabetic nephropathy (DN) is one of the most devastating microvascular complications of diabetes mellitus (DM) and is a common cause of end-stage renal disease (ESRD)^1,2^. The mortality rate of DN has risen rapidly in the last two decades and its prevalence has been increasing globally. Currently, DN therapy is limited to a few classes of drugs such as renin-angiotensin-system (RAS) blocker, glucagon-like peptide-1 (GLP-1) receptor agonist and sodium glucose transporter-2 (SGLT-2) inhibitor^3,4^. However, these candidate drugs have severe adverse reactions or poor therapeutic effects and there are no highly effective first-line treatment options^5,6^. Therefore, new therapeutic target molecules or cellular processes are needed as additional treatment options. Although many studies have reported the treatment of DN, only a few have focused on the treatment of different stages of DN.

As previously reported, DN is characterized by the development of tubulointerstitial fibrosis (TIF) and impaired renal function^7–9^. Evidence proved that RTECs that underwent partial EMT had an over deposition of ECM after injury, and caused the formation of TIF^10–12^. Autophagy is vital in maintaining organ homeostasis and stress adaption. Impaired autophagy is frequently associated with renal damage^13–17^. Furthermore, insufficient autophagy is a major factor in renal injury in DN, and it may be initiated by a variety of cellular stressors such as starvation, hypoxia, and endoplasmic reticulum (ER) stress^18–20^. It was reported that autophagy is influenced by EMT-related signaling pathways. Conversely, the activation of autophagy could suppress or strengthen EMT by regulating relevant signaling pathways^21^. Essentially, inhibition of the mammalian target of rapamycin (mTOR) has been shown to activate autophagy while suppressing proliferation and EMT in thyroid cancer cell lines^22^. Moreover, the activation of AMPK signaling has been proved to attenuate renal injury by suppressing partial EMT and chemokine secretion of chemokines in RTECs^23^. However, it remains unclear whether activating autophagy in RTECs will inhibit the progression of EMT and thus prevent DN.

Metformin, a first-line drug for the treatment of type 2 diabetes, was found to be effective in lowering blood glucose with minimal side-effects^24^. The activation of the signaling enzyme AMPK is one of the most extensively studied mechanisms for metformin^25,26^. Under hyperglycemic conditions, RTECs inactivated AMPK and autophagy, and activated mTOR, EMT, and hypoxia^8,27^. In this study, the potential effects of metformin on AMPK pathways during different stages of DN were explored in the study.

**Scheme 1:**
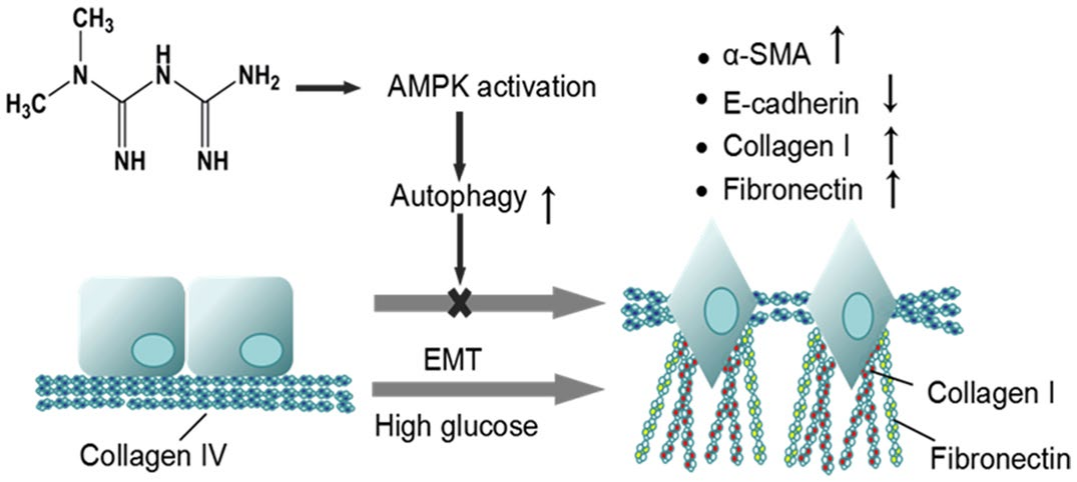
Metformin blocks the epithelial changes associated with partial EMT. Normal epithelial cells show strong cell–cell adhesion and have a basal matrix consisting primarily of type IV collagen and laminin (left). Upon induction of partial EMT under high glucose condition, the cells lose their adhesion and change morphology (right). These cells cleave and invade the basal lamina, followed migrating along a newly formed matrix containing fibronectin and type I collagen. The abundance of epithelial biomarker (E-cadherin) is reduced, while that of mesenchymal marker (α-SMA) is increased.

We hypothesized that metformin could prevent partial EMT and TIF by enhancing AMPK-induced autophagy in different DN stages (Scheme 1). The hypothesis was tested in streptozotocin (STZ)-induced DN rats and the underlying mechanisms were investigated in HG-stimulated RTECs. Our results demonstrated that metformin can reverse the partial EMT of RTECs stimulated by HG and TIF in DN rats via regulating autophagy, providing new insights on both the treatment of TIF and the relationship between EMT and TIF.

## Results

### Metformin improves renal function and downgrades blood glucose in DN rats

Blood glucose and biomarkers of renal function (BUN and SCr) were measured to evaluate the pharmacodynamics of metformin at various stages of DN. As shown in Table 1, there were no significant differences detected within the Sham groups, but conspicuous discrepancies between the 12-week (end stage) and the 4-week (early stage) were observed in the DN group (*P* < 0.05). Compared to the Sham group, only blood glucose revealed obvious differences in the 4-week DN group. However, significant differences were noted in blood glucose, BUN, and Scr in the 12-week DN group (*P* < 0.05). Blood glucose levels in the 4-week DN group decreased after metformin treatment, but they did not return to the normal. When compared to the end stage of DN, it was discovered that treatment with metformin in the early stage ameliorated blood glucose, BUN, and Scr to varying degrees (*P* < 0.05). These findings revealed that metformin had a better preventive effect in the early stage of DN. But as the disease progresses, the role of metformin in delaying the development of DN may be limited.

**Table 1:** Blood glucose and renal function of rats in each group (*n* = 6).

Group	Time (w)	Number	Blood glucose (mol/L)	BUN (mmol/L)	SCr (μmol/L)
Sham	4	6	6.05 ± 0.82	9.06 ± 1.25	38.31 ± 3.02
	12	6	5.95 ± 0.85	9.39 ± 1.08	40.61 ± 4.13
DN	4	6	25.00 ± 1.92*	9.97 ± 1.43	44.95 ± 6.17
	12	6	30.80 ± 2.01*^$^	14.61 ± 1.62*^$^	56.71 ± 6.45*^$^
DN + Met	4	6	16.48 ± 1.13*^#^	9.38 ± 1.80	39.53 ± 4.72
	12	6	19.12 ± 1.13*^#^	12.44 ± 1.73*^△^	47.08 ± 5.43*^#^

All data were shown as mean ± SD (*n* = 3).

BUN: blood urea nitrogen; SCr: serum creatinine; Sham: control group; DN: diabetic nephropathy group; Met: metformin.

*P < 0.01 vs. Sham group by ANOVA, ^#,△^*P* < 0.01 vs. DN group by ANOVA, ^$^*P* < 0.05 vs. 4-week DN and DN + Met group by ANOVA.

### Metformin attenuates renal injury in DN rats

To estimate the potential effects of metformin on attenuating renal injury, the body weight (BWT), ratio of KW/BWT and 24-h urine protein were measured. The BWT of the DN group was sharply reduced, although the KW/BWT ratio and 24-h urine protein were increased in both the 4-week and 12-week DN groups (*P* < 0.05). In addition, the BWT of the 12-week DN group was further decreased, whereas the KW/BWT ratio and 24-h urine protein were increased more significantly than in the 4-week DN group (*P* < 0.05). Renal dysfunction may develop from the renal injury of DN rats and aggravate with the progression of DN. Remarkably, metformin was found to be more effective at improving the impaired renal function, especially in the early stages of DN (Table 2).

**Table 2:** Comparison between BWT, KW/BWT, and 24-h urine protein in each group (*n* = 6).

Group	Time (w)	Number	BWT (g)	KW/BWT (%)	24-h urine protein (mg)
Sham	4	6	496.67 ± 19.67	0.59 ± 0.07	11.37 ± 1.57
	12	6	532.68 ± 28.68	0.57 ± 0.03	8.59 ± 2.00
DN	4	6	365.33 ± 19.00*	1.16 ± 0.14*	66.37 ± 6.86*
	12	6	332.17 ± 27.81*	1.81 ± 0.13*^$^	97.66 ± 9.76*^$^
DN + Met	4	6	429.83 ± 23.84*^#^	0.88 ± 0.06*^#^	51.99 ± 2.54*^△^
	12	6	396.33 ± 31.02*^#^	1.48 ± 0.24*^#^	82.73 ± 9.28*^#^

All data were shown as mean ± SD (*n* = 3).

BWT: body weight; KW: kidney weight; Sham: control group; DN: diabetic nephropathy group; Met: metformin.

*P < 0.01 vs. Sham group by ANOVA, ^#,△^*P* < 0.01 vs. DN group by ANOVA, ^$^*P* < 0.05 vs. 4-week DN/DN + Met group by ANOVA.

### Metformin improves renal morphology

**Figure 1:**
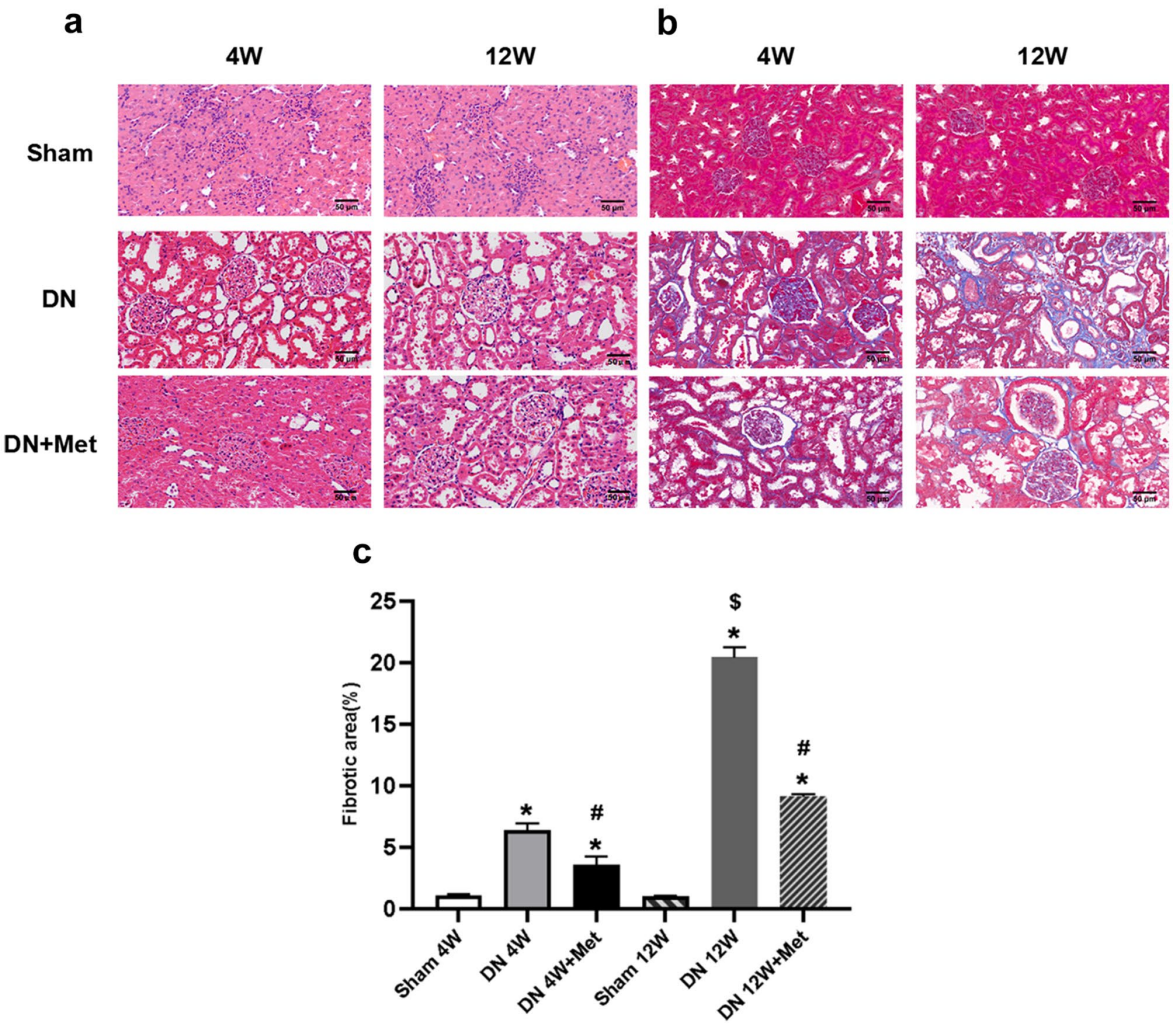
Effects of metformin on renal histology and extracellular matrix expansion. (**a**) Renal histology based on H&E staining. Scale bar represents 50 μm. (**b**) Extracellular matrix expansion based on Masson staining. Scale bar represents 50 μm. (**c**) Statistical analysis of renal fibrotic areas in each group. *P < 0.01 vs. Sham group by ANOVA, ^#^*P* < 0.01 vs. Sham and 4-week DN group by ANOVA, ^$^*P* < 0.05 vs. 4-week DN/DN + Met group by ANOVA. 4W: 4-week; 12W: 12-week; Met: metformin.

Throughout the experiment, H&E staining of the Sham group showed negligible fibrosis. However, severe renal damages appeared in both the 4-week and 12-week DN groups, including glomerular hypertrophy, mesangial matrix hyperplasia, renal tubular dilatation, and inflammatory cell infiltration. The impaired morphological changes in the 4-week DN group were relieved and tended to normalize after metformin treatment (Fig. 1a). Furthermore, the pathological changes of vacuolar degeneration, interstitial widening, interstitial fibrosis, and ECM over deposition in the DN groups appeared to be significantly more severe in the 12-week group than in the 4-week group. The blue areas were shrunk noticeably as metformin was used, indicating that metformin could reduce ECM over deposition and the fibrotic areas throughout the DN process, especially in the early stages (Fig. 1b,c). These findings demonstrated that metformin had a crucial role in kidney protection and TIF scavenging by attenuating both collagenous fiber and ECM over-deposition.

### Metformin lowers the expression of fibronectin and collagen I

**Figure 2:**
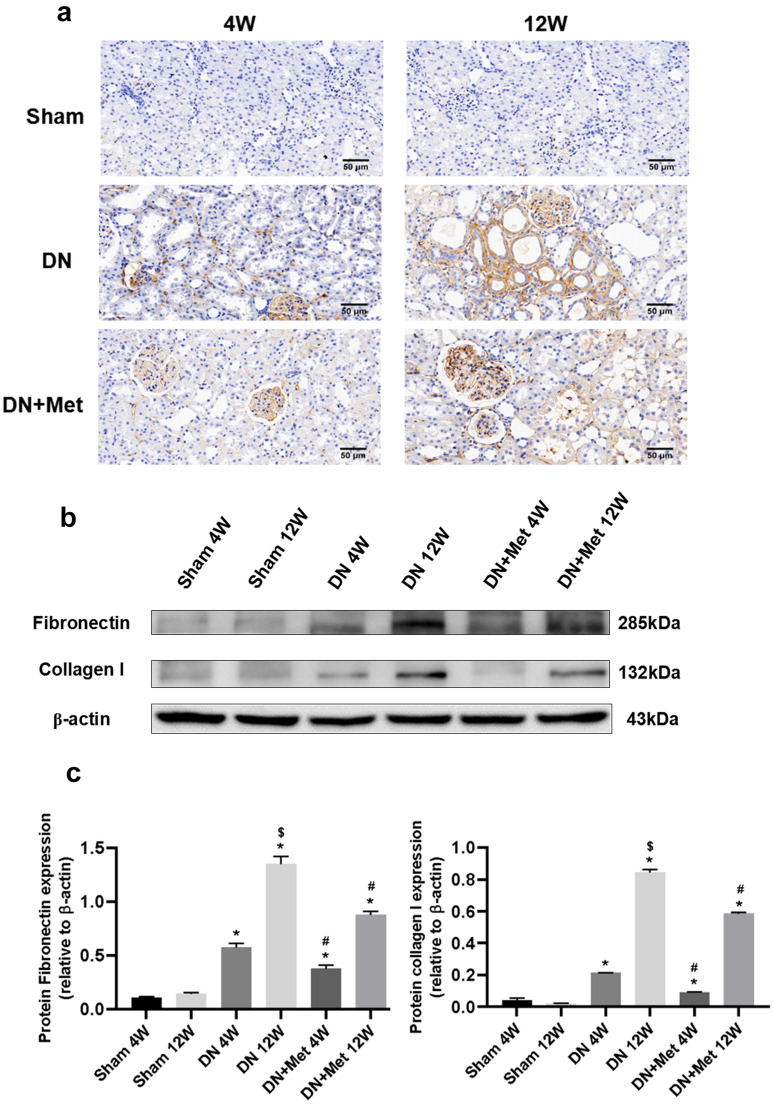
Effects of metformin on expression of FN and collagen I in DN kidneys. (**a**) Collagen I was observed by immunohistochemistry under a microscope. Scale bar represents 50 μm. (**b**) Expression of FN and collagen I based on immunoblotting and normalized to β-actin. The boxes separate images from different gels, but samples derived from the same experiment and blots were processed in parallel. (**c**) Statistical analysis of the expression of FN and Collagen I in each group. *P < 0.01 vs. Sham group by ANOVA, ^#^*P* < 0.01 vs. Sham and 4-week DN group by ANOVA, ^$^*P* < 0.05 vs. 4-week DN/DN + Met group by ANOVA. 4W: 4-week; 12W: 12-week; Met: metformin. Full-length blots were presented in Supplementary Figures.

Collagen I and fibronectin (FN) are two major constituents of extracellular matrix in the kidney. To further confirm the varieties of fibrosis, the expression of collagen I and FN was assessed. Collagen I was quantified using immunohistochemistry and immunoblot, whereas only immunoblot was used to measure FN. Collagen I expression was widely distributed in the DN kidney, especially in the late stages of the disease but its deposition was reduced and distribution restricted after treatment with metformin. Furthermore, the expression of collagen I was downgraded more evidently, and the therapeutic effects were more distinguished in the early stages of DN (Fig. 2a). These findings were corroborated by the immunoblotting results of FN and collagen I. The expression of FN and collagen I proteins was considerably higher in the 12-week DN group than in the 4-week DN group, indicating that the fibrotic situations of DN were getting severe as the disease prolonged (Fig. 2b,c). The scavenging activity was significantly higher after 4 weeks of metformin treatment than after 12 weeks, which may be attributed to the effect of metformin on delaying the formation of fibrosis at an early stage rather than reversing the progression of renal fibrosis throughout the process.

### Metformin improves the level of autophagy

**Figure 3:**
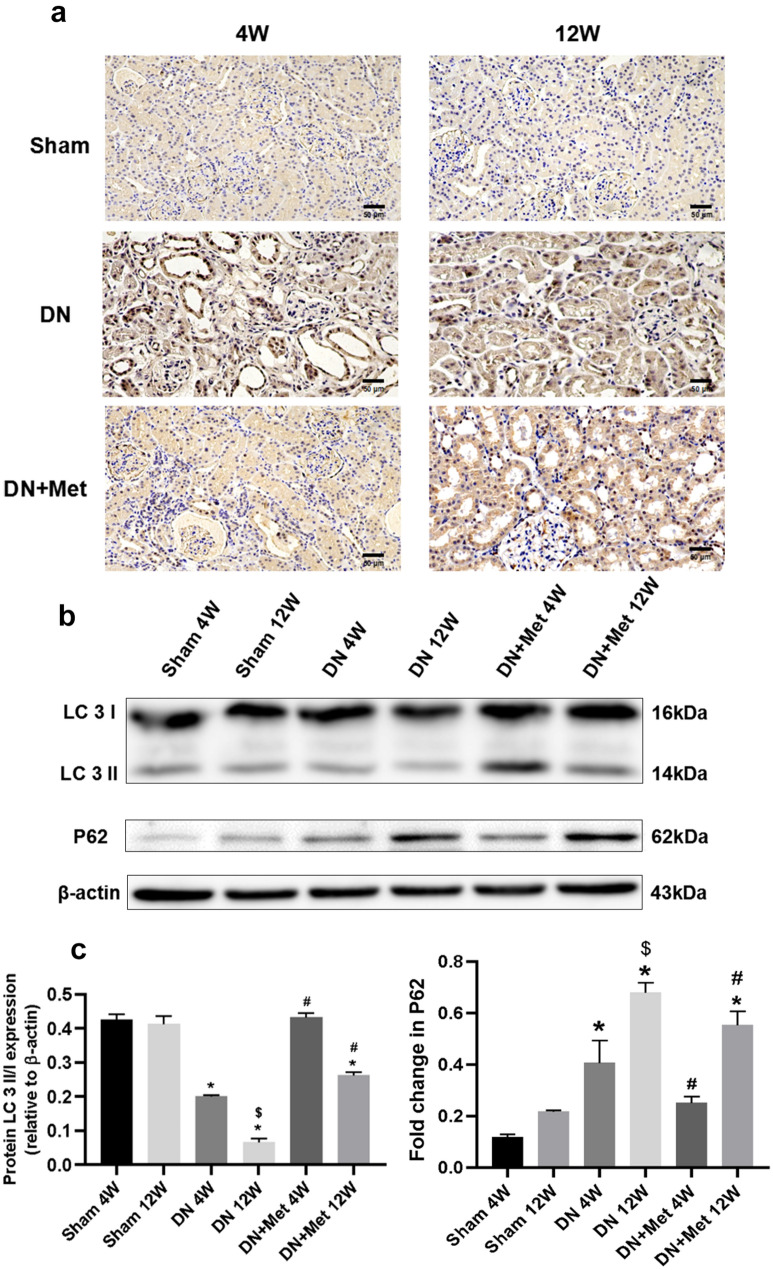
Effects of metformin on the level of autophagyin DN kidneys. (**a**) The level of P62 was measured using immunohistochemistry under a microscope. Scale bar represents 50 μm. (**b**) Expression of LC3 I and LC3 II based on immunoblotting and normalized to β-actin. The boxes indicate images from different gels, but the samples derived from the same experiment and blots were processed in parallel. (**c**) Statistical analysis of the expression of P62 and LC3 II/LC3 I in each group. *P < 0.01 vs. 4-week Sham group and 12-week Sham group by ANOVA, ^#^*P* < 0.01 vs. 4-week DN group and 12-week DN group by ANOVA, ^$^*P* < 0.05 vs. 4-week DN/DN + Met group by ANOVA. 4W: 4-week; 12W: 12-week; Met: metformin. Full-length blots were presented in Supplementary Figures.

The immunohistochemistry results showed the expression of the autophagy flux marker P62. The expression of P62 implied a high level of autophagy under normal conditions, but it increaed significantly in the 12-week DN group. However, after the treatment of metformin, the expression of P62 was downgraded, although the effect was relatively weak in the 12-week group (Fig. 3a). According to the LC3 II/I analysis, the ratio was lower in the early stages of DN than in the Sham group, along with a further decline in the late stages of DN (Fig. 3b). Consistent with previous results, the expression of P62 was up-regulated under the DN condition, and metformin could alleviate the P62 levels in the DN groups, especially in the 4-week DN group, confirming the effect of metformin on amelioration of autophagy (Fig. 3b,c).

**Figure 4:**
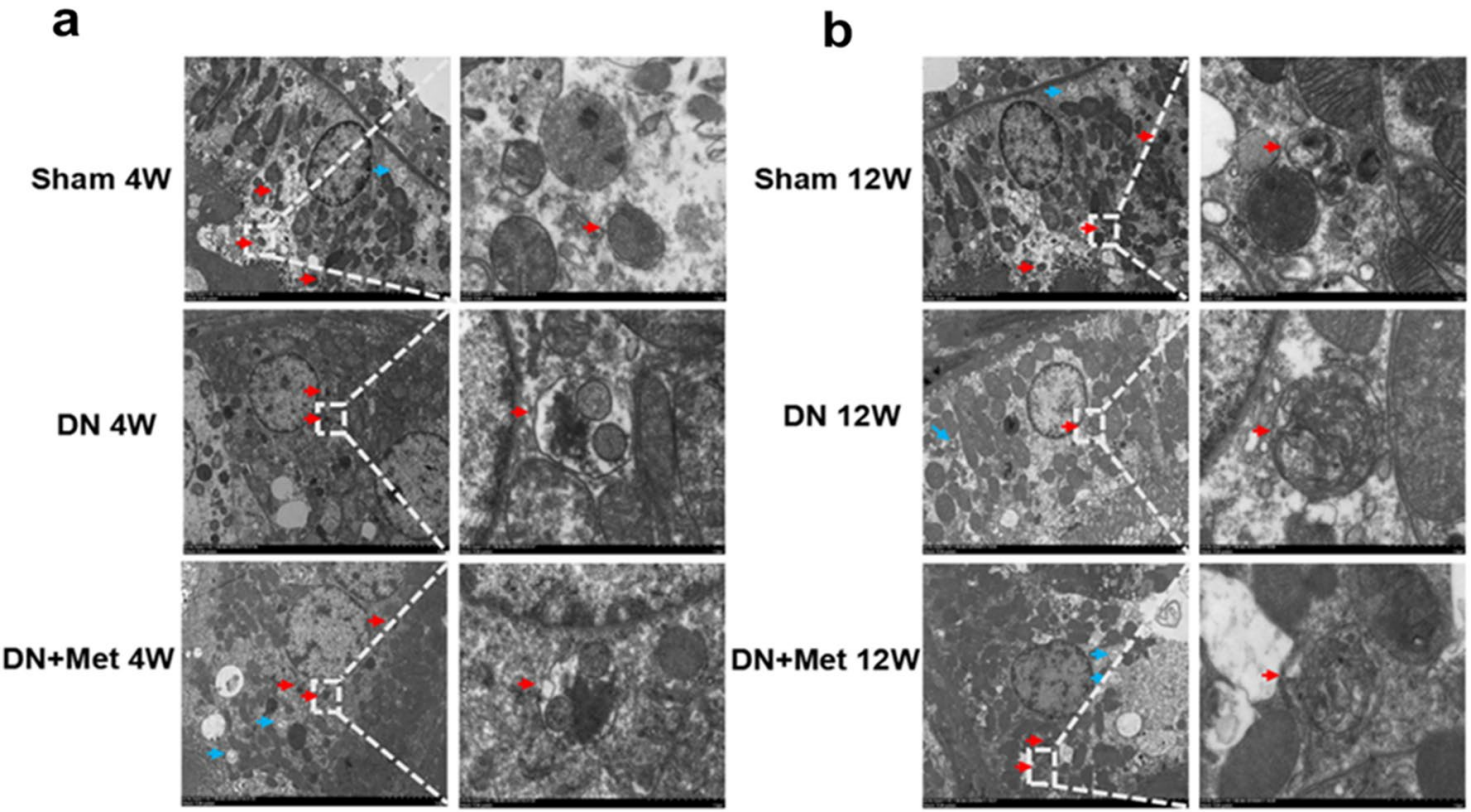
Effects of metformin on autophagy and autolysosome in DN kidneys. Autolysosomes were detected by transmission electron microscope at the 4-week molding (**a**) or 12-week molding (**b**). The blue arrow indicates autophagy, and the red arrow indicates autolysosome. Scale bar represents 5 μm (left) and 1 μm (right) respectively. 4W: 4-week; 12W: 12-week; Met: metformin.

The double membrane autolysosome is a hallmark of autophagy, and it can be distinguished clearly in an electron micrograph of normal RTECs. Furthermore, the mitochondrial cristae, autophagosomes, and autophagic lysosomes have distinct edges in the cytoplasm. The results showed that the autolysosomes in the DN group were reduced when compared to the Sham group, which was followed by morphological changes. The morphological changes were getting worsened as the DN prolonged, and the distinct clear boundary gradually disappeared. However, the morphological changes and the decrease of autolysosomes were reversed in both the 4-week and 12-week DN groups with the treatment of metformin, indicating that metformin may enhance the level of autophagy in the entire DN process (Fig. 4a,b).

**Figure 5:**
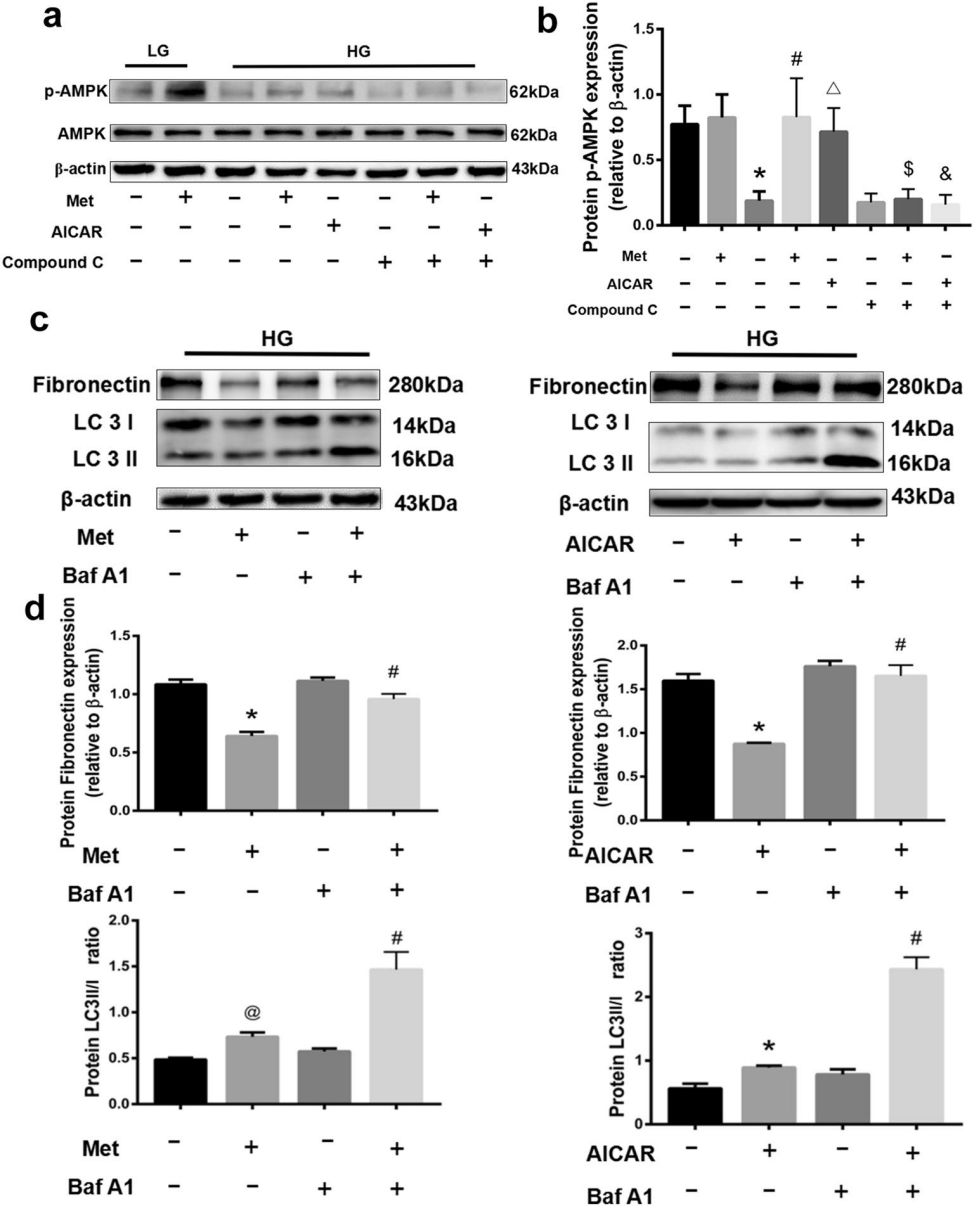
Effects of metformin on the expression of p-AMPK, LC 3I, LC 3II and FN in HG-stimulated RTECs. (**a**) Metformin and AICAR upgraded the p-AMPK expression in HG-stimulated RTECs. The boxes indicate images from different gels. (**b**) Statistical analysis of p-AMPK expression in each group. *P < 0.01 compared with the LG group, ^#^*P* < 0.01 compared with the HG without metformin group, ^△^*P* < 0.01 compared with the HG group, ^$^*P* < 0.01 compared with HG + Met group, ^&^*P* < 0.01 compared with HG + AICAR group. (**c**) Metformin positively regulated the ratio of LC 3II/I and downgraded the expression of FN, while Baf A1 negatively regulated the ratio of LC 3II/I and upgraded the expression of FN. The boxes indicate images from different gels, but the samples derived from the same experiment and blots were processed in parallel. (**d**) Statistical analysis of FN expression and the ratio of LC 3II/I in each group. *P < 0.01 compared with the HG without metformin and Baf A1 group, ^#^*P* < 0.01 compared with the HG with Baf A1 but without metformin group. Full-length blots were presented in Supplementary Figures.

The relationship between autophagy and fibrosis was further explored in vitro experiments. The expression of p-AMPK showed a significant decrease under the stimulation of HG, which could be effectively reversed by metformin (*P* < 0.05). Notably, metformin had comparable boosting effects on the p-AMPK as the well-known AMPK-activator AICAR, and the effect was weakened by the AMPK-inhibitor compound C, implying that metformin might efficiently activate AMPK signaling pathway (Fig. 5a,b). In addition, the ratio of LC 3II/I in HG-stimulated RTECs was upgraded and the expression of FN was downgraded after the treatment with metformin, but these effects were significantly blocked by the autophagy inhibitor Baf A1 (Fig. 5c). Parallel results were obtained when metformin was replaced by AICAR, indicating that metformin potentially improves cellular autophagy by activating the AMPK signaling pathway to minimize renal fibrosis (Fig. 5d).

### Metformin prevents partial EMT via AMPK activation

**Figure 6:**
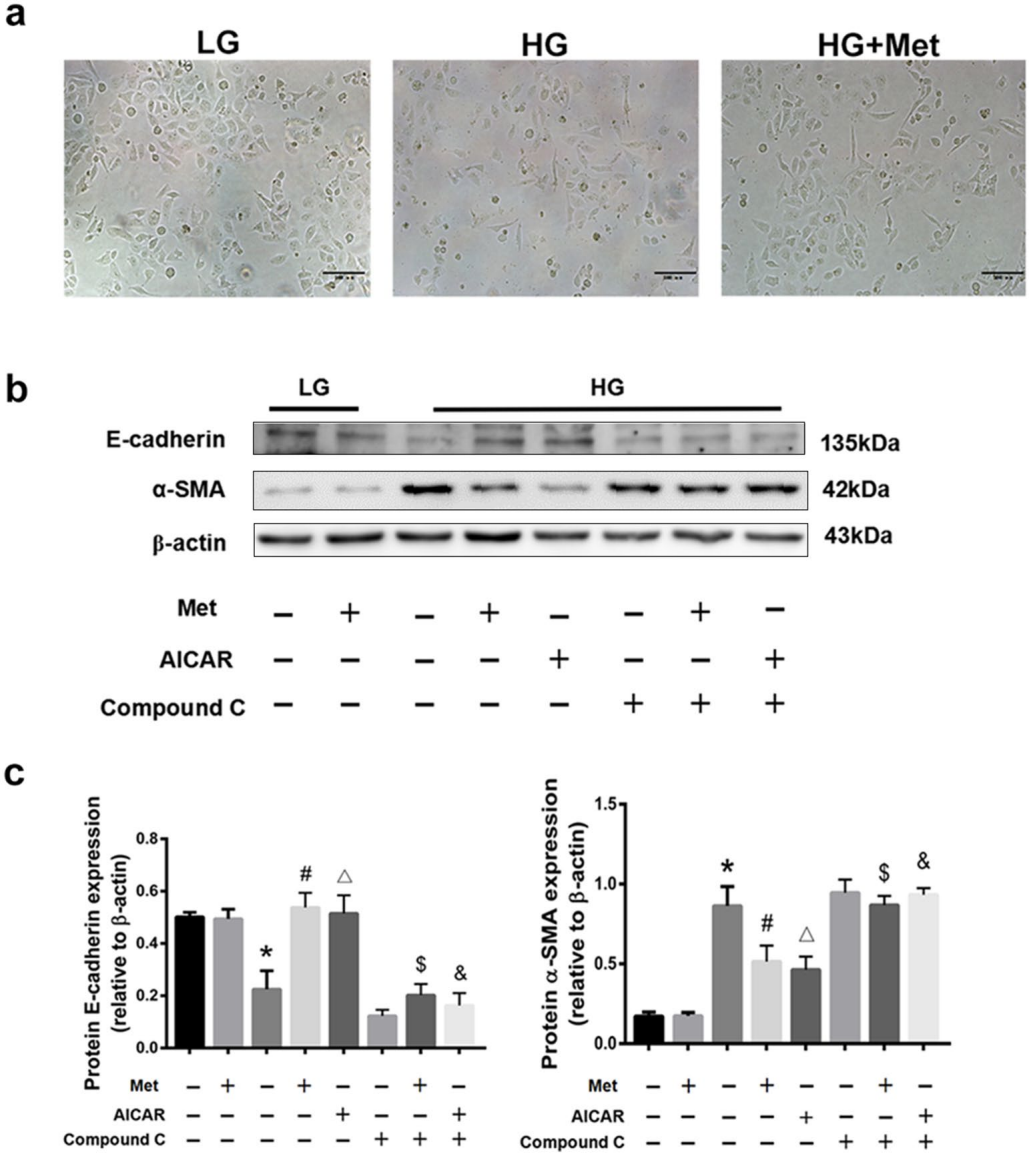
Effects of AMPK activators and inhibitors on the partial EMT of HG-stimulated RTECs. (**a**) Morphological changes of RTECs under different environments. (**a**) Western blot analysis of E-cadherin and α-SMA after different treatments. The boxes indicate images from different gels, but the samples derived from the same experiment and blots were processed in parallel. (**c**) Statistical analysis of E-cadherin and α-SMA in each group. *P < 0.01 compared with the LG group, ^#^*P* < 0.01 compared with the HG without metformin group, ^△^*P* < 0.01 compared with the HG group, ^$^*P* < 0.01 compared with HG + Met group, ^&^*P* < 0.01 compared with HG + AICAR group. Full-length blots were presented in Supplementary Figures.

To confirm the mechanism of metformin within the partial EMT, AICAR (an AMPK activator) and compound C (an AMPK inhibitor) were recruited. High glucose-stimulated RTECs exhibited growth inhibition and cellular morphological changes, from a cobblestone-like to spindle-like shape, but these effects were reversed by the treatment of metformin (Fig. 6a). We detected the expression of two well-known EMT markers, E-cadherin and α-SMA in RTECs. Immunoblotting of the RTECs in LG group showed the expression of E-cadherin was high whereas α-SMA was low regardless of whether metformin existed or not (Fig. 6b). However, the expression of E-cadherin was down-regulated in the HG group, whereas the expression of α-SMA was up-regulated. We observed that using metformin reversed the afore-mentioned processes. This outcome was comparable to that of AICAR, although the therapeutic influence disappeared after the treatment with compound C, confirming that metformin may activate AMPK and prevent the partial EMT of RTECs in HG condition. In addition, when metformin (or AICAR) and compound C existed at the same time, EMT traits were in consistent with those in the HG group, which further indicated that activating AMPK induced the inhibitory effects of metformin on partial EMT(Fig. 6c).

## Discussion

Autophagy is a highly conserved catabolic process for the degradation of cytoplasmic components^28^. Hyperglycemia may inhibit autophagy in kidney tubules in diabetic animals and patients with diabetes mellitus. Studies have shown that autophagy was impaired in HG-stimulated RTECs, diabetic kidneys of mice and kidney biopsy samples from DN patients^29,30^. Autophagy appeared to be activated in RTECs as an adaptive response to excess protein caused by diabetes-induced proteinuria. However, excessive or prolonged albumin uptake may impair autophagy in RTECs through an mTOR-dependent mechanism, eventually leading to progressive tubular injury^31,32^. Our results were consistent with previous findings^9^. The levels of blood glucose and 24-h urine protein were elevated in varying degrees in both the 4-week and 12-week DN rats, which were associated with autophagy inhibition as characterized by a decrease in the LC 3II/I ratio and an increase in P62 expression. In HG-stimulated RTECs, the decrease of LC 3II/I ratio indicated that cellular autophagy was also downgraded. Furthermore, it was reported that basal autophagy in kidney cells is essential role for maintaining renal structure, homeostasis, and functionality. Interestingly, HG-stimulated RTECs showed obvious morphological changes from cobblestone-like to spindle-like shape. E-cadherin was a distinctive protein for renal tubular epithelial cells as well as a sensitive marker of EMT. Western blot analysis revealed that the expression of E-cadherin was down-regulated while the expression of α-SMA and FN was up-regulated, indicating that RTECs underwent partial EMT process and promoted TIF progression under the stimulation of HG. Autophagy plays a role in mediating the degradation of pernicious or dysfunctional cellular components. Therefore, we hypothesized that RTECs under HG-stimulated conditions would impede cellular autophagy and undergo partial EMT, leading to increased accumulation and decreased degradation of TIF.

Metformin is one of the most widely prescribed oral anti-diabetic medications, and recent research has proved that it is an effective AMPK activator^33^. On the one hand, our results demonstrated that metformin reversed morphological changes in HG-stimulated RTECs, increased p-AMPK and E-cadherin expression, and decreased α-SMA and FN expression. On the other hand, metformin increased the LC 3II/I ratio, an effect that was weakened by the use of autophagy inhibitor. These findings indicated that metformin can enhance cellular autophagy by activating AMPK, reversing the partial EMT process in HG-stimulated RTECs and finally down-regulating the expression of fibrotic proteins.

The afore-mentioned findings were validated by in vivo experiments. Substantial morphological changes, such as renal tubular atrophy and necrosis, were observed in all of the DN rats. Besides, immunohistochemistry and western blot results showed that TIF deteriorated with the progression of DN. By contrast, blood glucose, 24-h urine protein, the indicators of renal function, and the level of autophagy improved with metformin treatment, which also reversed the renal morphological changes to varied degrees and considerably ameliorated TIF.

Previous studies found that EMT-related signaling pathways can trigger or repress autophagy. Meanwhile, autophagy was also involved in the induction and inhibition of EMT^34^. However, the cellular mechanisms that drive interstitial fibrosis are still being debated. Assigning autophagy to tubular EMT in the progression of renal fibrosis played either an important or a negligible role^35,36^. More recent studies had shown that RTECs underwent a partial EMT that was crucial for disease progression, but RTECs did not directly contribute to the formation of the myofibroblast. Instead, RTECs lost their normal tubular function, and the damaged cells released paracrine signals into the renal interstitium, reshaping the microenvironment^37^. While there was controversy concerning the role of EMT in the development of different types of fibrosis, the evidence strongly suggested that EMT activation was essential for the development of several types of fibrosis^38^. Thus, it is meaningful to imperative the relationship between renal TIF and the partial EMT process of RTECs. Our results indicated that the partial EMT process of RTECs contributed to the progression of renal TIF. Metformin can increase autophagy the levels by activating the AMPK pathway to, reversing the partial EMT of RTECs and reducing renal TIF.

Although the results of this study showed that increase of autophagy reduced fibrosis the changes in the corresponding pathways were not explored. Bernard et al.^39^ showed that sustained autophagy activated mTORC2 signaling, leading to the secretion and enhanced expression of CTGF and the transformation of fibroblasts to myofibroblasts, favoring the development of fibrosis in a model of prolonged serum starvation of fibroblasts. Furthermore, in TGFβ1-treated primary human atrial myofibroblasts, autophagy was necessary for the induction of fibrotic signaling and the synthesis of both collagen 2 and FN^40^. These results inspired us to believe that the catabolic machinery of autophagy could participate in an anabolic process of protein synthesis, which became the target of our subsequent research.

Despite the fact that a large number of studies has explored the pharmacodynamics of metformin in various fibrosis, there are still few investigations on the therapeutic effects of metformin in various stages of DN. Our results revealed great differences in functional biochemical parameters between the 4-week DN group and 12-week DN group. Specifically, we initially measured the body weight and KW/BW of DN model rats at 4 weeks and 12 weeks to roughly reflect the effects of metformin on different DN stages of rats. The results showed that rats in the early stages of DN lost less weight, and had lower KW/BW. This phenomenon indicated that the pathological changes of rats became more severe with the progression of DN. To explore the changes in kidney function in rats at different stages of DN, we assessed several classic indices (24 h-urinary protein, BUN, SCr). By reflecting the changes of biochemical indicators of renal function, we discovered that the decrease of renal function was consistent with the aggravation of DN, i.e., as DN progressed, renal function continued to deteriorate, and the difference was obvious. Next, we studied the changes in the kidneys of DN rats to explore the causes of changes in renal function. According to H&E results, the end stage of DN had severe morphological changes than the early stage, including more substantial glomerular hypertrophy, mesangial matrix hyperplasia, renal tubule dilatation and inflammatory cell infiltration. Simultaneously, new signs of renal interstitial fibrosis, including vacuolar degeneration, interstitial widening, interstitial fibrosis and excessive ECM deposition appeared. In addition, the collagen I and FN immunohistochemical and immunoblot results confirmed that the level of renal fibrosis in the end stage of DN was significantly more severe than that in the early stages of DN. Interestingly, despite some differences from normal conditions, pathological changes were improved to varying degrees after metformin treatment, especially in the early stages of DN, which was reflected in the improvement of body weight indexes, the ratio of KW/BWT, renal function recovery, the weakening of renal morphological changes, and the reduction of renal interstitial fibrosis. To investigate the potential mechanism of metformin, we explored the effects of metformin on autophagy and EMT at different DN stages. The results implied that metformin activated AMPK throughout the DN process and regulated autophagy flux and the number of autophagosomes, thereby increasing the level of autophagy in DN. Furthermore, in the HG-stimulated RTECs, metformin activated the AMPK pathway to attenuate partial EMT. The effects of increased autophagy and reduced EMT were more obvious in the early stages of DN, while they were relatively weak in the later stages.

A possible explanation for these phenomena may be the function of metformin in the TIF. Metformin may play a role in preventing future lesions in healthy cells or tissues, rather than reversing the diseased parts back to the initial state. Our results showed that the pathological changes were minimal in the early stages of DN. Therefore, metformin can protect primarily healthy cells or tissues from further deterioration, resulting in more significant therapeutic effects on 4-week DN. However, when DN progressed to the end stage, the therapeutic effects were limited because metformin could not reverse the formed lesions, although its use was still effective.

In summary, the present study demonstrated that metformin protected the renal TIF of STZ-induced diabetic rats. These beneficial effects could be mediated by autophagy activation and EMT suppression. The induction of autophagy provided by metformin was successful in treating TIF, especially in the early stages of DN. The results provided new therapeutic options for diabetic renal TIF, as well as a foundation for further research into the relationship between EMT and TIF.

## Conclusion

Metformin attenuated diabetic renal TIF via activating AMPK-induced autophagy and suppressing partial EMT of RTECs, notably in the early stages of the disease. Our study provides a theoretical and experimental foundation for future research on the potential clinical application of metformin in the treatment of diabetic TIF.

## Methods

### Materials and reagents

Metformin was purchased from Sigma-Aldrich (St Louis, USA). Dulbecco’s modified Eagle medium (DMEM) was obtained from Gibco (Massachusetts, USA) and fetal bovine serum (FBS) was purchased from ScienCell (California, USA). The Cell-Counting Kit 8 (CCK8) was provided by Dojindo (Shanghai, China). The other chemicals were of analytical grade and used without additional purification. Primary antibodies against AMPK, FN, collagen I, LC I, LC II, α-SMA, P62 and E-cadherin were obtained from Abcam (Cambridge, UK). Primary antibody against β-actin was bought from Proteintech Group (Chicago, USA).

### Cell culture

The RTECs were supplied by the Cell Bank of the Chinese Academy of Sciences (Shanghai, China) and cultured in Keratinocyte Serum-Free Medium (K-SFM) containing 1% penicillin/streptomycin at 37 °C and 5% humidity. For stimulating the condition of renal fibrosis, RTECs were administrated with 30 mmol/L HG for 48 h. To explore the effects of metformin on the HG-stimulated RTECs, 1 mol/L metformin was added into the medium for 24 h. Compound C (10 mmol/L) was pretreated for 18 h before AICAR (1 mol/L) was added in corresponding groups for 24 h. Metformin (1 mol/L) was co-cultured with Baf A1 (5 mmol/L) in HG-stimulated RTECs.

### Experimental animals

Male SD rats weighing 200–250 g (6–8 weeks) were purchased from the Laboratory Animal Centre of Xuzhou Medical University (Xuzhou, China). All experiments involving animals were performed in line with the animal use protocol approved by the Institutional Animal Care and Use Committee of Xuzhou Medical University (permit number: SYXK2015-0030). In addition, this study was carried out in accordance with the ARRIVE guidelines. All methods followed the relevant guidelines and regulations.

The animal experiments were conducted based on the original procedure with minor adjustments^41^. The rats in the Sham group (12 rats) were fed with a standard diet whereas those in the DN group (24 rats) were fed with a high-glucose and high-fat diet (18% lard, 20% sugar, 3% egg yolk, and 59% standard diet). Besides, the rats in the DN group were given a single intraperitoneal injection of 1% STZ solution (50 mg/kg). Blood samples were taken 72 h after injection, and blood glucose ≥ 11.1 mmol/L were used as primary models. After additional another 4 weeks of feeding, the 24-h urine protein was measured and values > 150% of normal were defined as successful DN models. Metformin was injected intraperitoneally at a dose of 65 mg/kg/day, and the rats in the DN group were intraperitoneally injected with the same volume of normal saline.

### Biochemical measurements

A blood glucose monitor was used to detect blood glucose levels after a 12-h of fast. The level of the blood urea nitrogen (BUN) and serum creatinine (SCr) were measured using assay kits purchased from Jiancheng Bioengineering Institute (Nanjing, China).

### Histology

The kidney specimens were first immobilized in 4% formaldehyde before embedding in paraffin and sectioned at a thickness of 3 μm. Then kidney tissue sections were dyed with hematoxylin–eosin and Masson's trichrome stain (Solarbio, Beijing, China) in line with the vender's instructions.

### Immunohistochemistry

The kidney tissue sections were baked in a 65 ℃ oven, deparaffinized in xylene, hydrated in ethanol, dipped in the boiling citric acid buffer (pH 6.0), then treated with 3% hydrogen peroxide to inactivate endogenous peroxidase activity. The slides were incubated with the primary antibodies, anti-FN (1:100), and anti-LC3 II (1:100), at 4 °C overnight after being blocked in 1% BSA for 1 h. After three washes with PBS, the diaminobenzidine (DAB) was added as the substrate, and the nuclei were counterstained with hematoxylin.

### Western blot

The samples were lysed on ice for 30 min in RIPA and PMSF (RIPA:PMSF = 100:1), then centrifuged at 12,000 rpm for 15 min at 4 °C. Approximately 150 μg of total protein was loaded onto sodium dodecyl sulfate–polyacrylamide (SDS) gels after boiling for 10 min, and transferred to a polyvinylidene difluoride (PVDF) membrane by electroblotting. For specific binding, membranes were blocked in 10% milk for 2 h at room temperature and then incubated overnight at 4 °C with corresponding primary antibodies against LC 3A/B (1:1000), Collagen I (1:800), FN (1:800), P62 (1:1000), E-cadherin (1:1000), α-SMA (1:800), AMPK (1:800) or β-actin (1:1000). Afterwards, the PVDF membranes were incubated with corresponding secondary antibodies for 1 h at room temperature. The signal was quantified by β-actin, visualized with ImageQuant LAS 4000 mini and measured using gray value using Image J software (GE, USA).

### Transmission electron microscopy

The kidney tissues were dehydrated in a graded series of ethanol after being fixed in 2.5% glutaraldehyde fixative overnight. Then the specimens were subsequently sliced into 50–70 nm thick slices with an ultrathin microtome, stained with lead citrate and uranyl acetate, and examined using a HT7700 TEM (HITACHI, Japan).

### Statistical analyses

In this study, data analysis was performed using SPSS 21.0 software. All data were expressed as mean ± SD. Differences among groups were analyzed using one-way analysis of variance (ANOVA), and the comparisons between groups were assessed using t-tests. *P* < 0.05 was considered statistically significant.

## Supplementary Information

Supplementary Information.
