# Supplementary material for: Metformin attenuates renal tubulointerstitial fibrosis via upgrading autophagy in the early stage of diabetic nephropathy
Source: Sci Rep. 2021 Aug 11;11:16362. doi: 10.1038/s41598-021-95827-5 (PMC8357942; doi:10.1038/s41598-021-95827-5)
Supplement: Supplementary file 1 — Supplementary Information. [file 41598_2021_95827_MOESM1_ESM.pdf]

Supplementary material of "Metformin attenuates renal tubulointerstitial fibrosis via upgrading autophagy in the early stage of diabetic nephropathy" (ID c9bc53e8-5506-497b-85a7-88a00d8ce0e4).

The un-cropped wider scans of fibronectin (FN) and Collagen I in Figure 2b:

FN

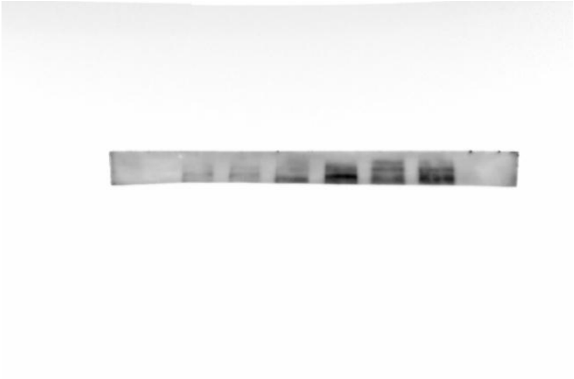

(Original image)

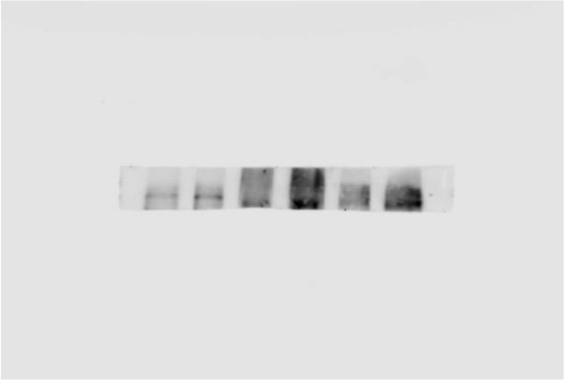

(Different exposure 1)

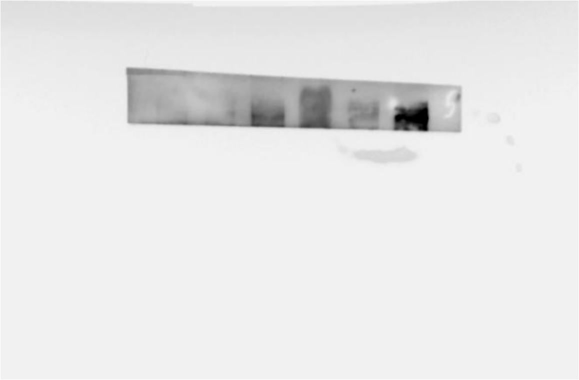

(Different exposure 2)

Collagen I

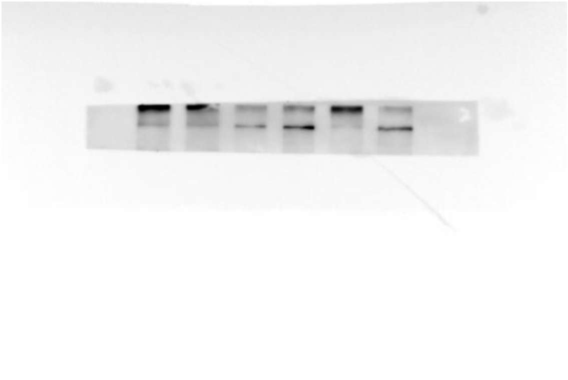

(Original image)

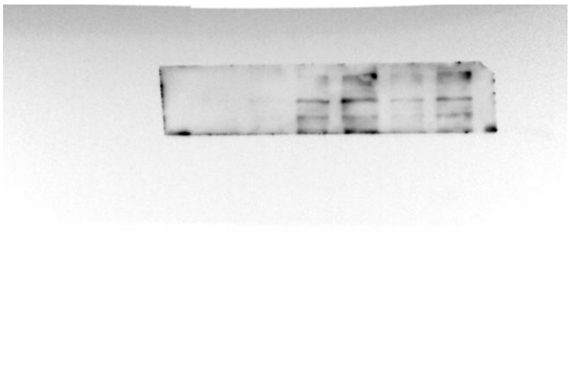

(Different exposure 1)

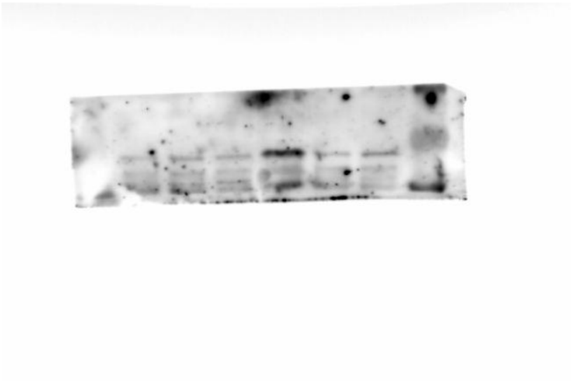

(Different exposure 2)

The un-cropped wider scans of LC 3 II and LC 3 I and P62 in Figure 3b:

LC 3 II and LC 3 I

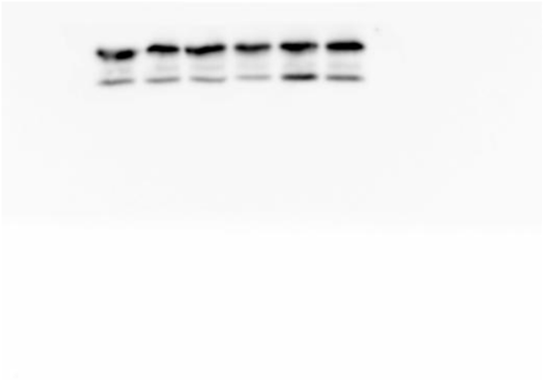

(Original image)

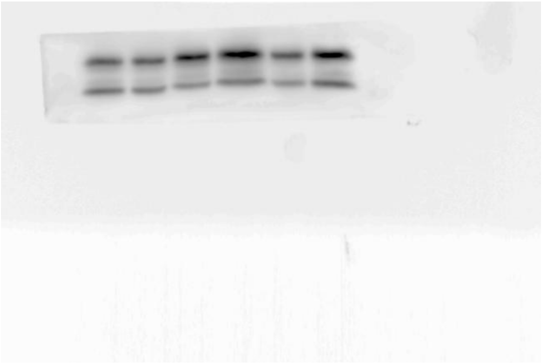

(Different exposure 1)

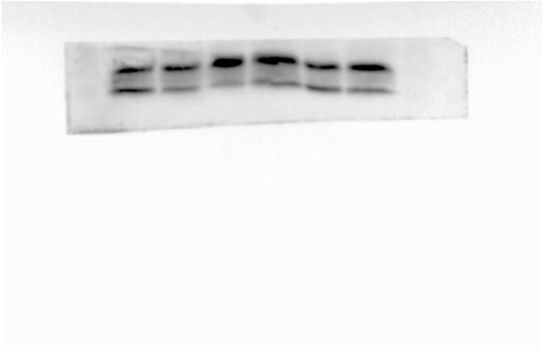

(Different exposure 2)

P62

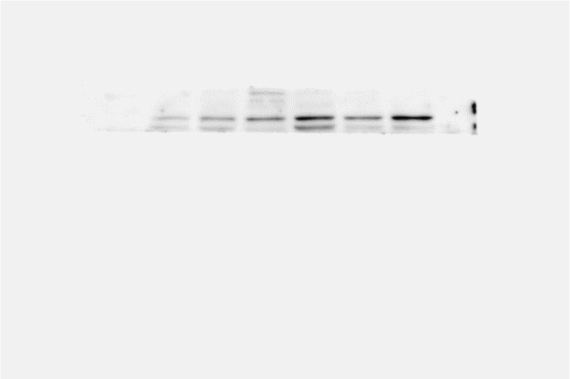

(Original image)

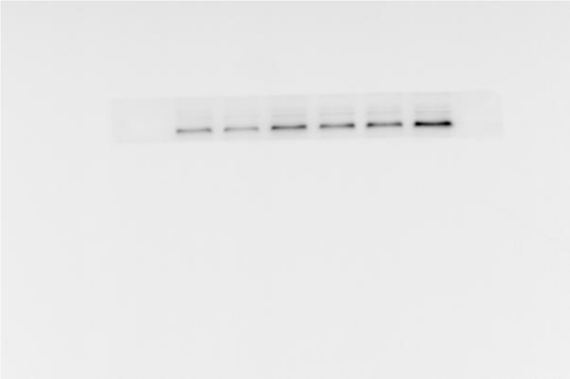

(Different exposure 1)

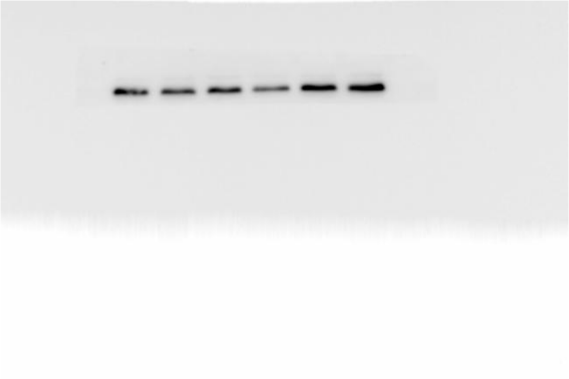

(Different exposure 2)

The un-cropped wider scans of p-AMPK andAMPK in Figure 5a:

p-AMPK

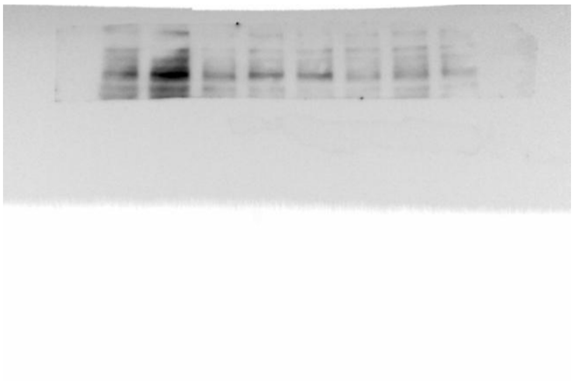

(Original image)

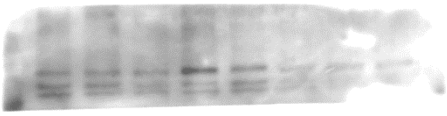

(Different exposure 1)

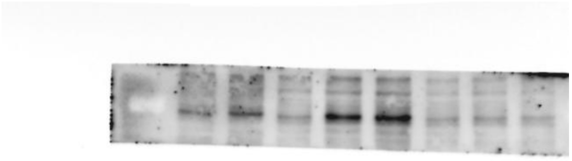

(Different exposure 2)

AMPK

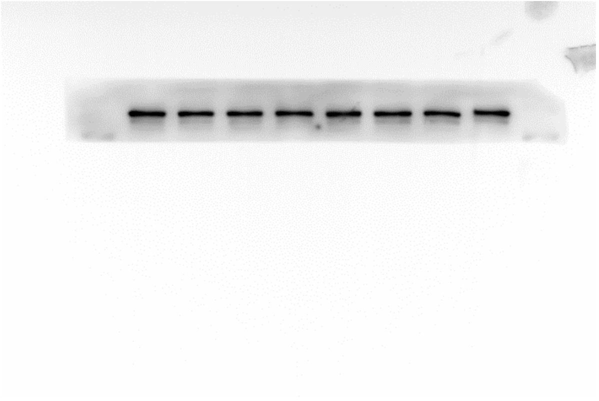

(Original image)

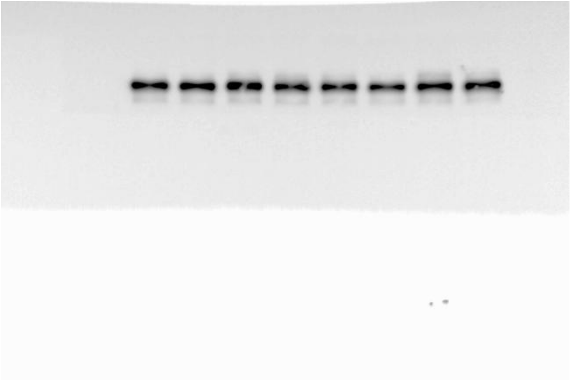

1(Different exposure 1)

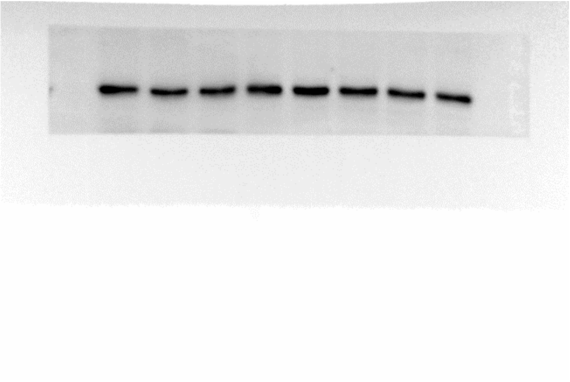

(Different exposure 2)

The un-cropped wider scans of LC 3 II and LC 3 I and fibronectin (FN) in Figure 5c:

FN (left):

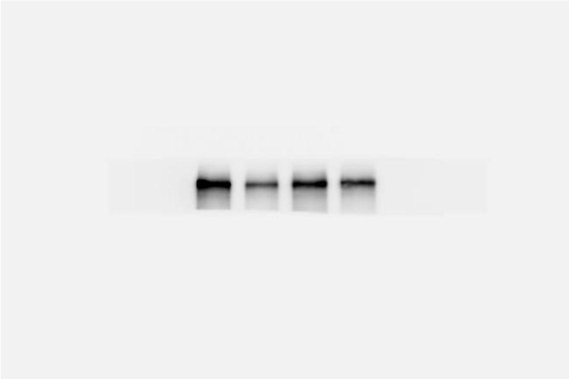

(Original image)

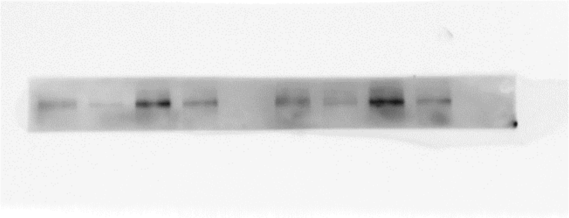

(Different exposure 1 and 2)

FN (right):

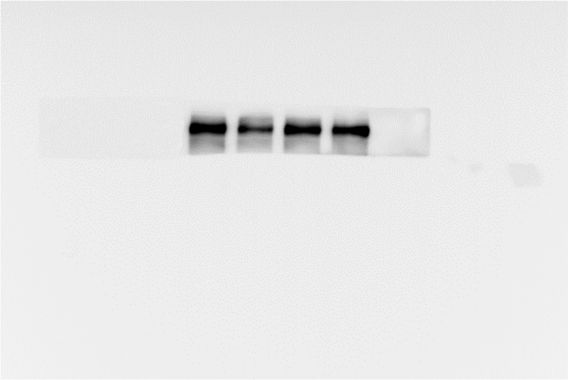

(Original image)

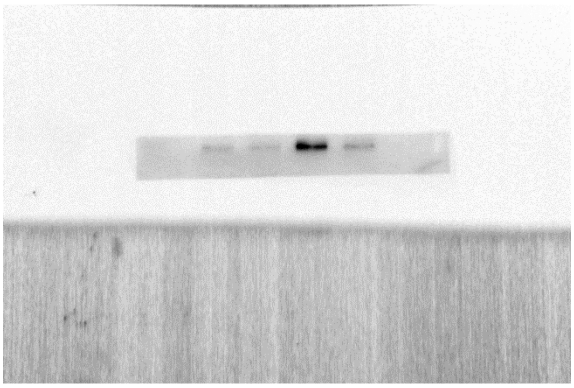

(Different exposure 1)

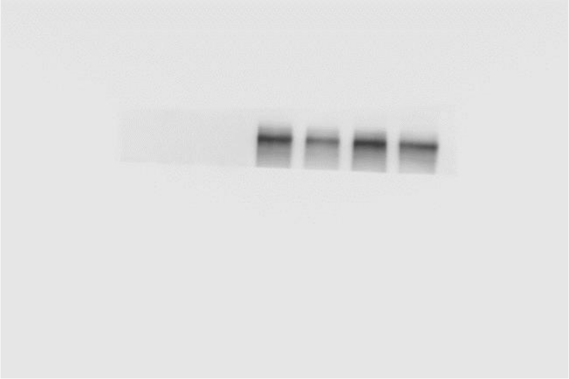

(Different exposure 2)

LC 3 II and LC 3 I (left):

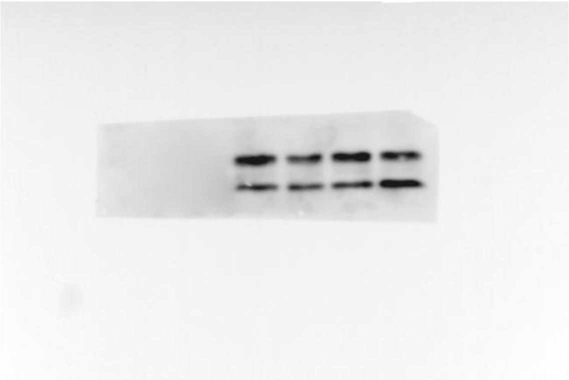

(Original image)

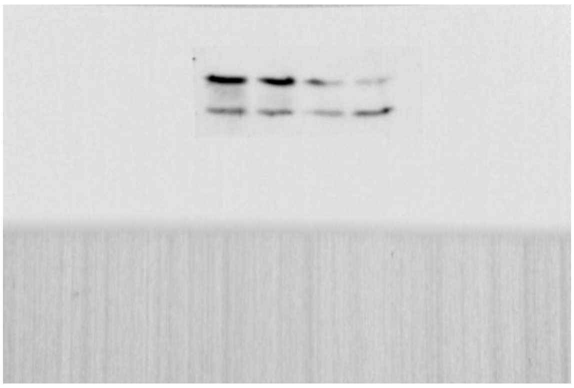

(Different exposure 1)

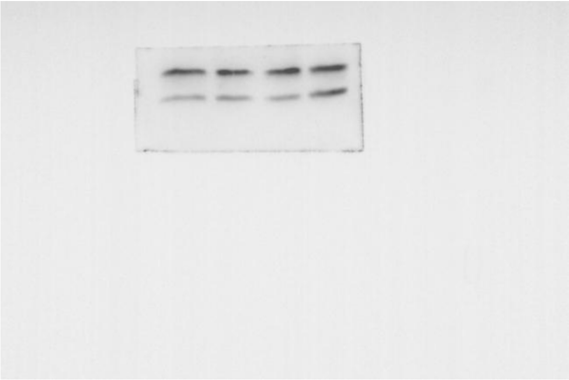

(Different exposure 2)

LC 3 II and LC 3 I (right):

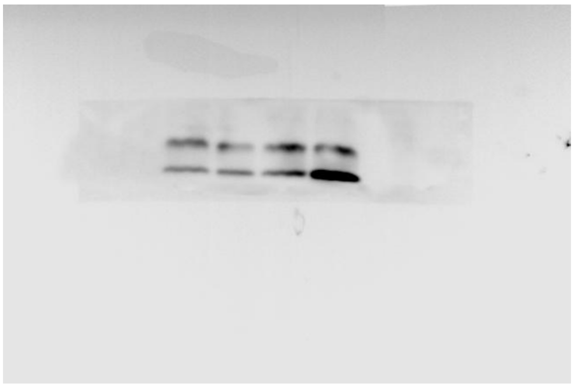

(Original image)

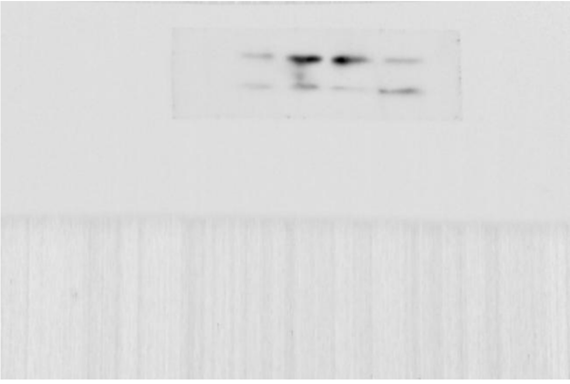

(Different exposure 1)

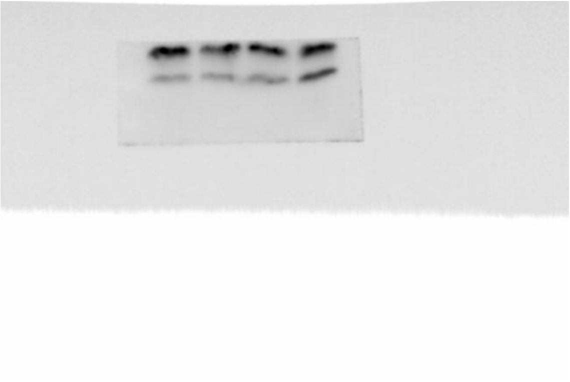

(Different exposure 2)

The un-cropped wider scans of E-Cadherin and α-SMA in Figure 6b:

E-cadherin

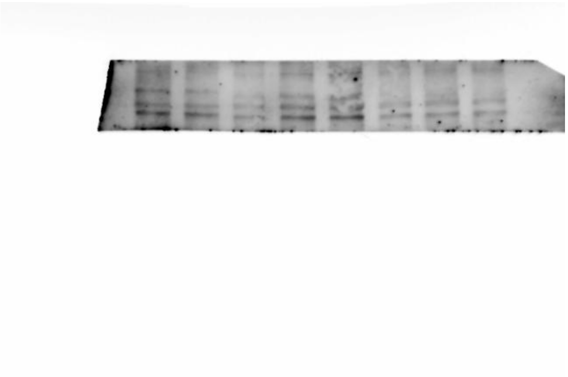

(Original image)

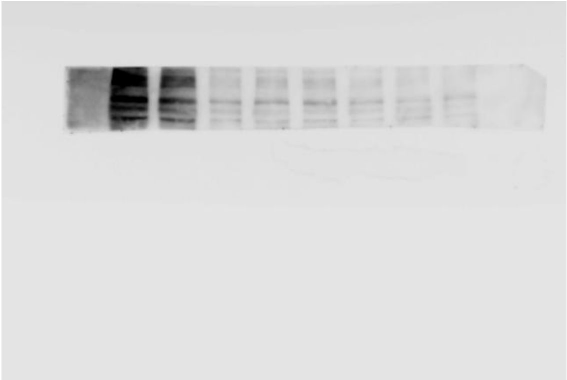

(Different exposure 1)

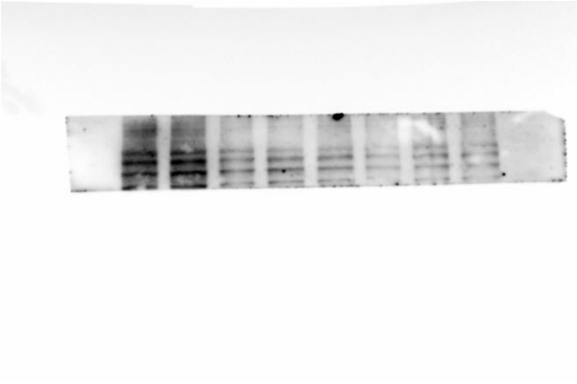

(Different exposure 2)

α-SMA

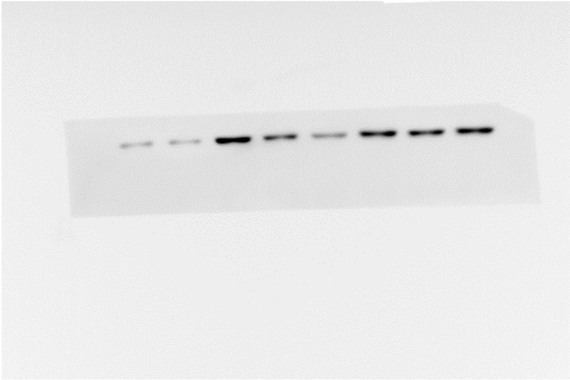

(Original image)

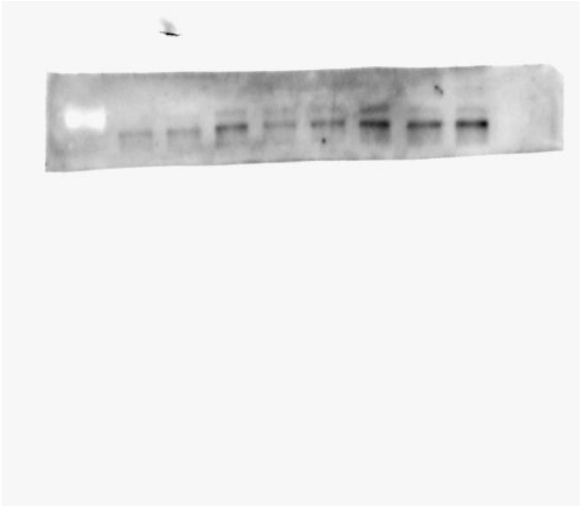

(Different exposure 1)

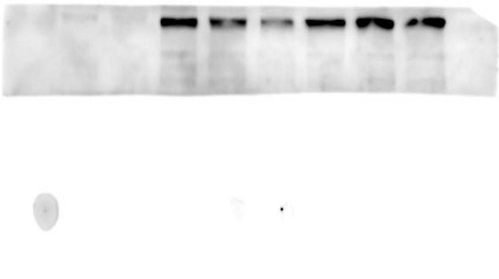

(Different exposure 2)

The un-cropped wider scans of β-actin in Figure 2b:

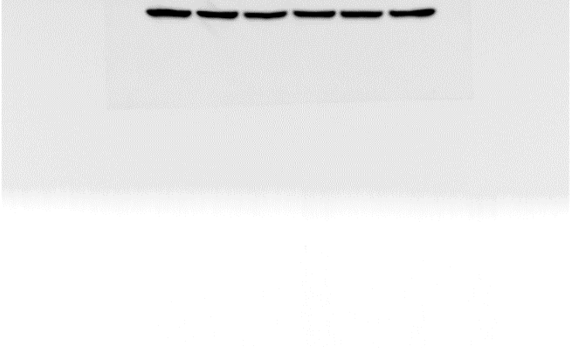

(Original image)

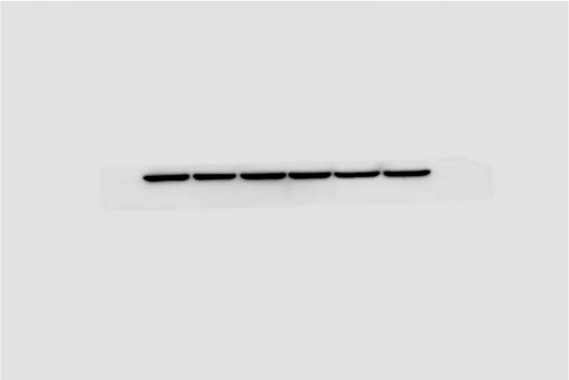

(Different exposure 1)

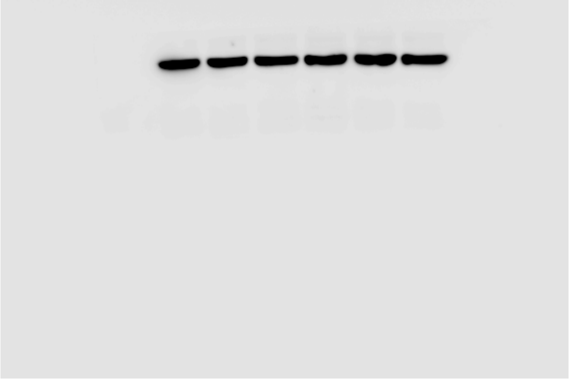

(Different exposure 2)

The un-cropped wider scans of β-actin in Figure 3b:

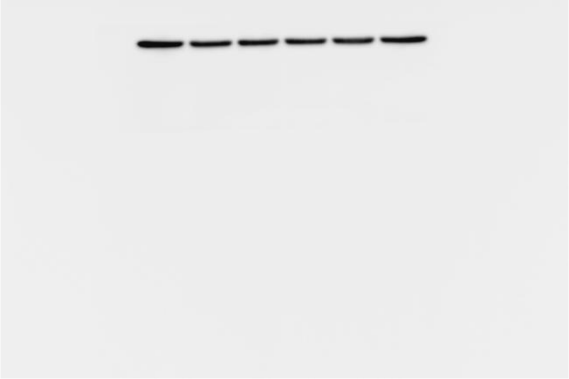

(Original image)

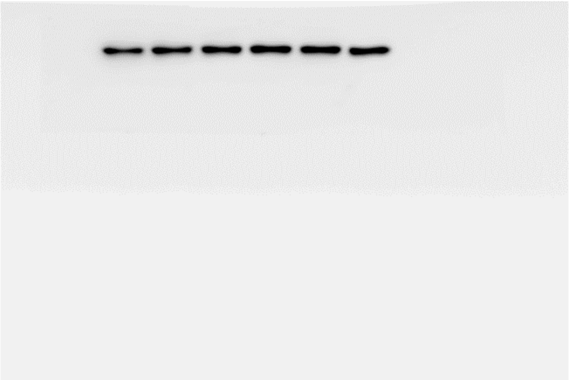

(Different exposure 1)

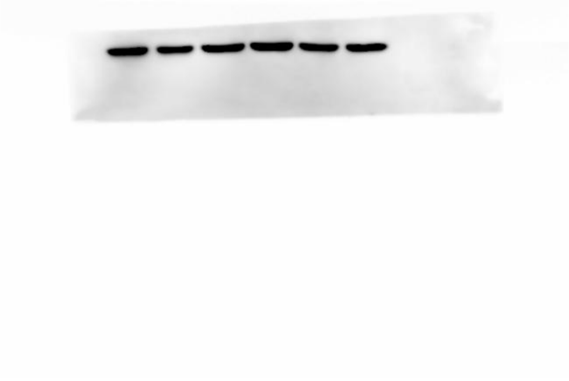

(Different exposure 2)

The un-cropped wider scans of β-actin in Figure 5a:

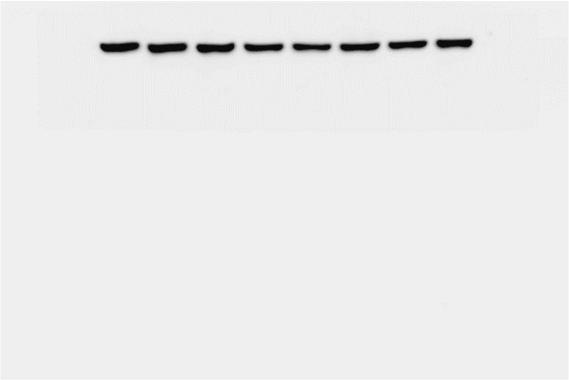

2(Original image)

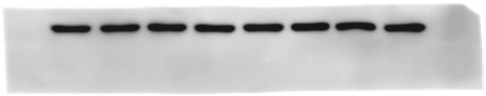

(Different exposure 1)

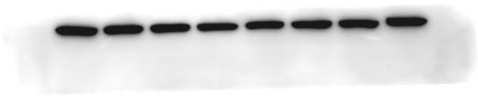

(Different exposure 2)

The un-cropped wider scans of β-actin (left) in Figure 5c:

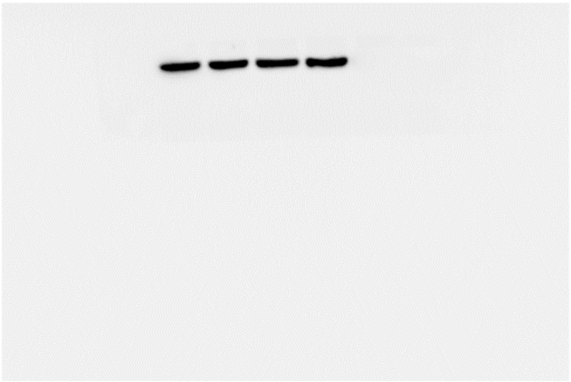

3(Original image)

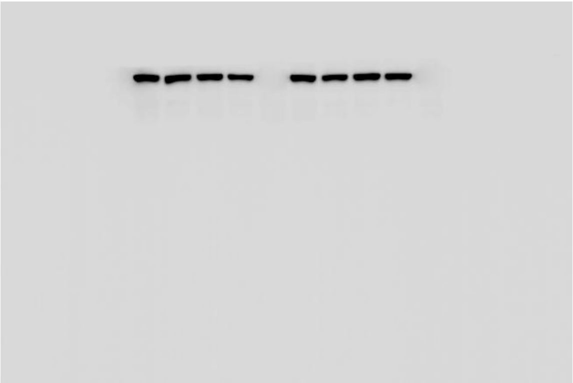

(Different exposure 1 and 2)

The un-cropped wider scans of β-actin (right) in Figure 5c:

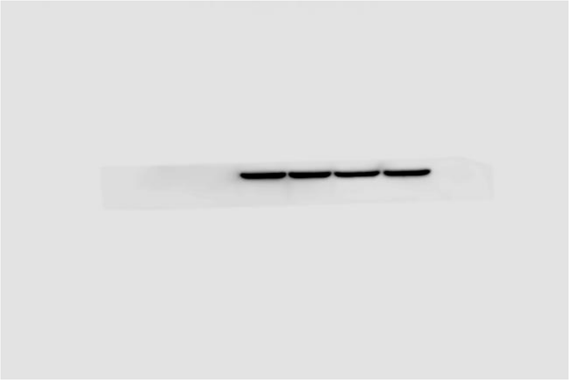

(Original image)

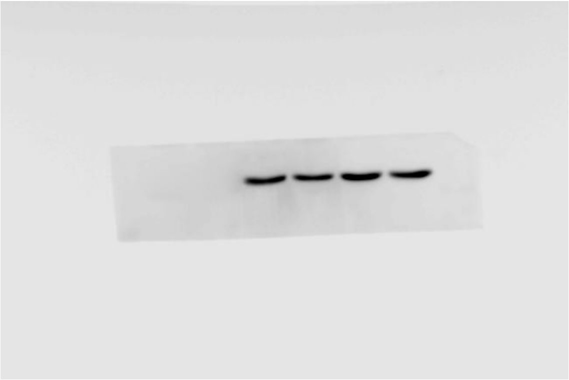

(Different exposure 1)

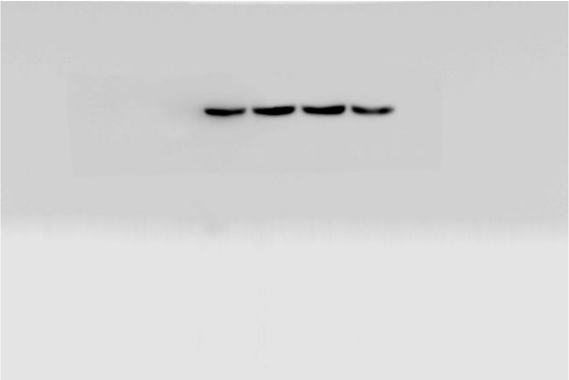

(Different exposure 2)

The un-cropped wider scans of β-actin in Figure 6b:

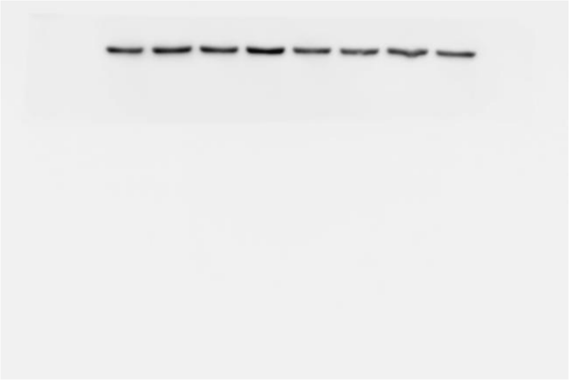

(Original image)

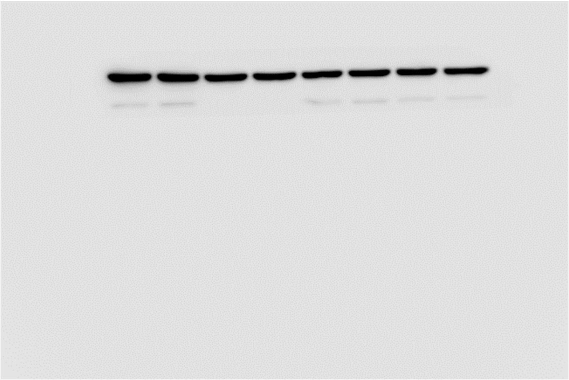

(Different exposure 1)

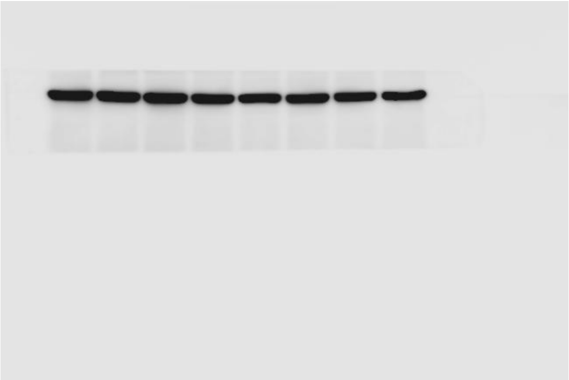

(Different exposure 2)
